# Acceptability and usability of assistive equipment and technology by individuals with multiple sclerosis: A qualitative study with occupational therapists

**DOI:** 10.1177/03080226241253765

**Published:** 2024-05-22

**Authors:** Courtney Barnett, Angela Murphy, Daniel Cezar da Cruz

**Affiliations:** MSc Occupational Therapy (Pre-Registration) Programme, School of Health, Leeds Beckett University, Leeds, UK

**Keywords:** Multiple sclerosis, assistive equipment and technology, occupational therapy

## Abstract

**Introduction:**

Assistive equipment and technology (AE&T) is often part of occupational therapy practice for individuals with multiple sclerosis (MS). We aim to explore the acceptability and usability of AE&T by people with MS from the perspective of occupational therapists.

**Methods:**

Our study applied a qualitative descriptive design, using semi-structured interviews conducted with five qualified occupational therapists with at least 6 months of experience in providing occupational therapy to adults with MS. Reflexive thematic analysis was used for data analysis.

**Findings:**

Three developed themes describe the occupational therapists’ experiences: ‘The cognitive impact of the illness rather than the physical’, ‘There’s a drive to not let the MS win’ and ‘They have to experience it by doing’. Therapists perceive cognitive changes, the meaning of technology and a person-centred approach as elements influencing the acceptability and usability of AE&T.

**Conclusion:**

Our findings highlight the importance of using core occupational therapy principles and approaches to best support people with MS in using AE&T effectively.

## Introduction

Multiple sclerosis (MS) is one of the most prevalent, unpredictable and progressive conditions causing neurological disability (BMJ Best Practice, 2021). MS affects around 2.8 million people worldwide, and over 110,000 people are estimated to be living with MS in England (MS International Federation, n.d.; PHE, 2020). These estimations have increased by 30% since 2013, along with a rise of newly diagnosed people each year (Walton et al., 2020).

MS is a demyelinating condition causing neural damage to the central nervous system (CNS; BMJ Best Practice, 2021). Although the aetiology remains unclear, research suggests genetic and environmental factors may be contributive (National Health Service, 2022). There are four main types of MS: (1) relapsing-remitting MS (RRMS), (2) primary progressive MS (PPMS), (3) secondary progressive MS (SPMS) and (4) benign MS. All these types can cause neurological symptoms that affect a person’s condition physically and cognitively (NICE, 2022).

People experience and manage the effects of MS uniquely to adapt to significant changes and life with the life-long degenerative condition (Cahill et al., 2016). This also includes a process of fear around dependence on assistive technology, such as wheelchair, which can progress to acceptance once individuals with MS experience how AE&T enables participation in certain occupations (Desborough et al., 2020). Occupational therapy plays a significant role in facilitating people’s participation in meaningful occupations (RCOT, 2019) to promote engagement in occupations people want to, need to or are expected to do (WFOT, 2012).

Examples of occupational therapy practice with people with MS includes providing rehabilitation, teaching compensatory strategies, managing fatigue and prescribing Assistive Equipment and Technology (AE&T) to assist and support people’s occupational participation (RCOT, 2019). AE&T refers to products or systems that facilitate and help individuals with a disability, restricted mobility or other obstacles to perform daily activities that might otherwise be impossible or difficult to perform (UK Government, 2021).

According to the World Health Organization (2018), there is a global need for AE&T because of an ageing global population. It is expected by 2030 that more than 2 billion people will need at least one product or assistive system. Although research suggests that AE&T is mostly used by people with disabilities or older adults (WHO, 2018), anyone could require AE&T over their occupational lifespan.

Evidence suggests that AE&T can provide a range of benefits to individuals living with MS, such as promoting occupational participation, reducing dependency on carers and improving quality of life (Squires et al., 2019). However, while there is evidence of AE&T usability in individuals with MS and others with different health conditions, such as stroke, the theme is not sufficiently studied (Cruz et al., 2016; de Joode et al., 2010). This has implications for the clinical and professional reasoning of prescribing equipment such as evidence-based practice. Thus, how usable and acceptable AE&T is and the complexities of its acceptance and usability require exploration because according to Arthanat et al. (2009), the usability of AE&T is a relevant indication of the individuals’ level of occupational participation in a range of occupations.

## Literature review

As already noted above, research on people with MS using AE&T is particularly scarce from an occupational ’therapist’s perspective. Despite the possible advantages of using AE&T for people with disabilities, there is evidence to suggest people with MS have had negative experiences associated with it, for reasons such as ‘feeling forced to try new devices’ (Squires et al., 2019: 486). There is a potential ethical dilemma which needs to be further understood regarding the negative feelings associated with AE&T.

Evidence focused on the use of AE&T reports difficulties in learning how to use it, retain its knowledge and manipulate it (Hedman et al., 2017). A literature review shows that the use of AE&T has a much broader focus on those with neurological conditions rather than those with the specific needs of the MS (Mortenson et al., 2018). Despite this limitation, the review emphasises the positive impact on carers, who reported feeling safer when AE&T was used. In some cases, it enabled a shift from providing physical assistance to supervision (Mortenson et al., 2018).

Boland et al. (2018) conducted a study focusing on users’’ perspectives on employing AE&T following a stroke. Their findings demonstrated a lack of knowledge and training regarding AE&T and the absence of follow-up after the introduction of AE&T. Training and follow-up of AE&T appear to be one of the reasons for abandonment reported in Cruz et al.’s (2016) study, therefore, challenging occupational therapists’ practices to maximise technology usability and to enable outcomes on individual occupational participation. In the UK, a focus group composed of MS individuals reported barriers related to the assessment and provision of assistive devices, including a mismatch between technology and the person’s needs (Tedesco Triccas et al., 2019). A recent Cochrane intervention protocol on MS affirms that despite the importance of occupational therapy with this population, its impact is still unknown (Kos et al., 2023).

Since occupational therapists assess and prescribe AE&T, they are more likely to have a greater insight into people’s reasons for not accepting AE&T and the experiences of its use in their daily lives. The AE&T abandonment raises questions about the usability of assistive technologies and how occupational therapists can support people with MS to participate in occupations using the technologies available. Moreover, taking into consideration the estimated rise in MS cases and potential demand for AE&T, our research aims to explore the identified gap in research on the usability of AE&T by individuals with MS from an occupational therapist’s perspective. This research sought to explore the following research question: what are the perceptions of occupational therapists who work with individuals with MS regarding the acceptability and usability of AE&T?

## Methods

### Study design

This research is situated within a naturalistic paradigm of inquiry where the world comprises several overarching realities that have subjective experiences, are constructed socially and change dynamically (Polatajko, 2004). A qualitative descriptive study was chosen to explore occupational therapists’ perspectives on the usability of AE&T by individuals with MS. The qualitative descriptive methodology is a generic and exploratory approach with no association with a specific theoretical orientation (Stanley, 2024: 52) although the positionality of the researchers as occupational therapists is relevant (Braun and Clarke, 2024). A descriptive approach is suitable for research in occupational therapy because it values the lived experience from the participants’ perspective (Stanley, 2024).

### Ethics

Our study was granted ethical approval by the Local Research Ethics Coordinator from Leeds Beckett University. Participants were informed that their participation in the study was voluntary, and they had the right to withdraw from the study at any time up to analysis. All participants received detailed information about the ’study’s aim and gave written and verbal consent. Participants received the interview schedule before the interview, giving them time to prepare for it (Yeo et al., 2023) and an opportunity to clarify any further questions or, for example, withdraw their participation. The following pseudonyms were used for each participant to ensure anonymity and confidentiality: Yumi, Sunita, Tarik, Sam and Leili.

### Participant recruitment and selection

Our descriptive study used purposive sampling as it intentionally recruited occupational therapists to describe their experiences with AE&T and individuals with MS. According to Stanley (2024), descriptive studies do not need representative sampling, but the researcher needs to inform on the details of the sample type. Hence, a purposive sampling of occupational therapists who have practised in AE&T with individuals with MS was selected to capture their singular lived experiences. The inclusion criteria comprised qualified occupational therapists with a minimum of 6 months experience, currently working with or recent experience of occupational therapy with adults with MS.

Following ethical approval, recruitment took place via social media. The study was advertised on established occupational therapy groups on Facebook, from a professional social media profile and with group administration permission. The advert also specified the inclusion criteria. Five participants met the criteria. Once data from these participants provided enough information to explore their perceptions regarding the acceptance and usability of AE&T by individuals with MS, data collection ceased. In addition, our study focused on the quality of data, since qualitative research is more concerned with meaning rather than generalisation of findings to a broader population (Carminati, 2018).

### Instrument and data collection

Semi-structured interviews were conducted to gather rich qualitative data from participants’ experiences. The duration of interviews varied from 45 to 60 minutes. The interview script comprised 13 open-ended questions, allowing time and opportunity for in-depth responses. Table 1 presents the interview questions.

**Table 1.** Interview questions.

Question	Description
1.	What are your experiences of working with people with MS?
2.	How is your experience of prescribing assistive equipment or technology?
3.	How do you describe any other assistive equipment or technology you have prescribed?
4.	What responses do you get from people with MS when prescribing assistive equipment?
5.	Do you perceive a difference in acceptance between smaller or larger equipment?
6.	What are the procedures after the assistive equipment has been accepted and prescribed?
7.	What do you do if a person with MS does not accept assistive equipment or technology?
8.	What do you think the risks or implications are of people with MS not accepting assistive equipment or technology?
9.	Are there any barriers to people with MS accepting assistive equipment?
10.	Are there risks involved to the person’s family, including carers?
11.	How to manage the challenges around refusal of equipment?
12.	What do you think needs to happen for people to use assistive equipment effectively?
13.	What advice would you provide to less experienced occupational therapists?

Interviews were carried out on video chat platforms, Microsoft Teams and Zoom in a reserved university room. Interviews took place in the participants’ own time and they all attended online and assured the researcher (CB) they were in confidential environments. Recorded interviews enabled the researcher to participate fully in the interviews. Spontaneous transcription and video recording also allowed revision of the transcripts to ensure accurate data representation and to increase credibility (Gray, 2018).

### Data analysis

Data collected from the interviews were transcribed verbatim, and reflexive thematic analysis – RTA was utilised inductively (Braun and Clarke, 2021, 2024) following reflection on research values as advised by Braun and Clarke (2024). This choice was made as it enabled the researchers to explore and interrogate the data, providing an in-depth description (Braun and Clarke, 2021). Following the six stages of RTA, transcripts were read and examined for semantic (surface, obvious, explicit) meaning (Braun and Clarke, 2021). The RTA process was conducted initially by the first author CB in consultation with AM (research supervisor), which involved discussions on possible themes and revisiting the data numerous times to define the themes. DC revised the literature, themes and discussion. Transcripts were coded by CB and themes were developed and refined by CB, DC and AM using direct quotations to capture the reflections and experiences of the participants. Themes were not predetermined but instead were identified through an organic and flexible approach. As with all qualitative research, the subjectivities of the researchers will have influenced interpretations of the data (Braun and Clarke, 2024). This was managed through reflexivity journals and attention to the experiences of the participants. Researcher interpretations are presented mainly in Discussion.

### Trustworthiness

The involvement of AM and DC supported the research trustworthiness (Nowell et al., 2017). CB set non-leading interview questions to limit and be alert to researcher influences with the support of AM. They were also able to recognise how their personal views might have influenced any interpretations and employed reflexivity throughout (as noted above). For transparency, CB and AM had previous professional clinical experience with individuals with MS and had observed reluctance and complexities in decision making when considering the use of AE&T in practice and DC had experience with AE&T research and clinical practice outside the UK. Reflexive journals were used to record reflections of personal views, pre-assumptions and experiences (Nowell et al., 2017) to ensure the process of analysis maintained a descriptive representation of the participants’ perspectives. To enhance the quality of the report the authors have followed the Reflexive Thematic Analysis Reporting Guidelines (RTARG) (Braun and Clarke, 2024) thus aiming to stay true to the appropriate quality markers of qualitative research and RTA and to the experiences of the participants. Direct quotations are considered to enhance confirmability and were used for the theme titles as well as within each theme as ways of encapsulating and illustrating the essence of what was said by the participants (Eldh et al., 2020).

## Findings

Participants’ experiences in providing occupational therapy to individuals with MS ranged from 5 to 26 years, with a mean of 18.8 (standard deviation 5.2 years). They worked in relevant specialist areas of occupational therapy practice, such as inpatient and outpatient services for adults, inpatient, community-based rehabilitation and specialist services for assistive technology provision.

Occupational therapists reported prescription of a range of AE&T interventions during interviews. These included environmental controls and adaptations (e.g. TV, heating, lights, radio, fan, telephone, alarms, bed and chair, door and window openers), perching stools. In addition, participants reported assistive or augmentative assistive communication aids, toileting and bathing equipment and adaptations (e.g. shower chairs, closomats for toileting, raised toilet seats, freestanding toilet frame, shower board), ground floor extensions, non-slip floors. Furthermore, mobility aids such as scooters, manual and powered wheelchairs, walking frames, hoists, banana boards, sara steadies, electric mini lifts, stairlifts, profiling beds and chairs. Pressure sore management air mattresses and specialist cushions, kitchen trollies, adapted cutlery as well as smaller aids, dressing aids, long-handled shoehorns, ergonomic setup at workstations, handwriting grips or aids, mobile arm supports, and mainstream technologies (tablets, mobile phones, computers).

**Figure 1.**
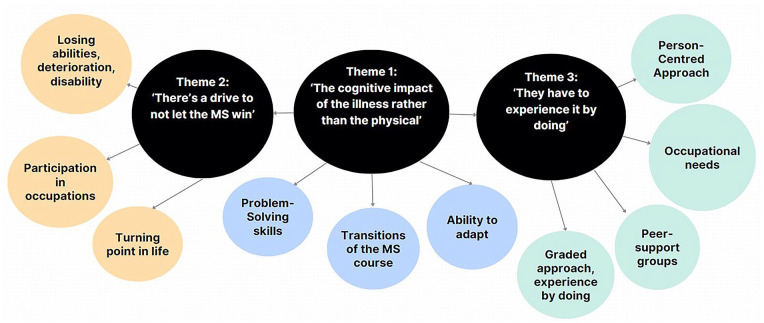
A thematic map of the three overarching themes on acceptability and usability of AE&T.

Figure 1 illustrates a thematic map with the three main intertwined themes: ‘Theme 1: The cognitive impact of the illness rather than the physical’, Theme 2: ‘There’s a drive to not let the MS win’ and Theme 3: ‘They have to experience it by doing’.

### Theme 1: ‘The cognitive impact of the illness rather than the physical’

Cognition was a topic in the discourse of each participant throughout the interviews. Yumi and Sunita shared their experiences of providing AE&T for adults with MS. They said how this health condition can have a significant impact on individuals’ cognition, for example, in problem-solving and processing information about AE&T:

One of the things that’s really common with people with MS is when there is cognitive involvement it’s often information processing that’s affected and problem-solving ability. (Yumi)

I think some people with MS do struggle to think flexibly, so they do struggle to think outside the box and think of other scenarios. (Sunita)

Leili and Tarik described the impact which MS can have on cognition as complex and dynamic, implying changes with the course of the disease. For these participants, having an awareness and knowledge of the cognitive issues and changes that occur for people with MS was as significant as understanding physical changes. However, they perceived the cognitive aspect as a significant barrier to the acceptance and usability of AE&T, with care needed in its presentation:

It is being quite aware about the cognitive deficits and changes, and I think. . . I see this as the biggest barrier more than the physical because, you can always compensate for the physical access. So, it’s the way, how you structure and how you present technology really. (Leili)

I think that’s my experience of why they don’t take well to the equipment to be honest, it’s more because of the cognitive impact of the illness rather than the physical. (Tarik)

Participants also explained that adults with MS may have difficulties in their adaptation of how to use new pieces of AE&T because it requires them to learn new strategies, which could be challenging for those experiencing cognitive impairment. As a result, Leili represented how changing the device impacted acceptance by an individual with MS:

Even though I was just changing the device, she wouldn’t have it. She wouldn’t accept the change because it operated a bit differently.

The context seems to influence acceptance and the usability of AE&T, which requires understanding the person’s circumstances. For instance, Yumi revealed that people learn to balance and manage their MS symptoms without the need for AE&T, up until a point where they no longer manage to participate in their occupations, whereby they are forced into acceptance technology:

So people are in this kind of balance where they actually can just manage with the situation as it is. But one thing changes at that point, and the whole house of cards falls down [. . .] So, it’s really interesting because you would look at them objectively as an outsider and think you need a wheelchair, you need to stay in this unit, you need this and that . . . her husband had developed dementia and he was admitted to hospital. And at that point, she wanted everything. So, we’d spent seven years trying to get her to have adaptations. And what happened was she ended up being stuck in one room with no access, toilet, shower, or kitchen. That was so awful. So now she’s desperate for these interventions. But we had been highlighting this to her for seven years, and she wouldn’t have it. And she said to me, she said to me the other day, I wished I got it.

The quotation above suggests that an acceptance of AE&T needs to be contextualised according to an individual’s unique circumstances. It appears that the ability to adapt with their own cognitive strategies to participate in occupations would be an influence on the acceptability of technology. Although in Theme 1 the impact of cognition, transitions of MS and ability to adapt was clear, in Theme 2, another perspective pointed out by occupational therapists indicates the emotional involvement with technology and its meanings as an important aspect related to acceptance and usability.

### Theme 2: ‘There’s a drive to not let the MS win’

Yumi stated an interesting point about understanding the difference between ‘insight’ and ‘acceptance’ and considered the impact of whether the individual fully understands their condition. Yumi’s discourse exposes that there is also the need to consider the meaning technology represents to the person, for example, an association with the finality of losing abilities that were expected to be regained or maintained, along with the harsh reality of living with a degenerative condition:

Most people will say something to you like ‘but if I start using a powered wheelchair, I’ll stop walking and then I’ll never walk again.’ But actually, their condition is leading them to that anyway. (Yumi)

Nonetheless, it is possible technology is also related to the stigma associated with having a disability. Yumi reported that individuals in their earlier stages of MS were more likely to accept AE&T if it appeared to look ‘normal’. Despite that, as the MS progresses and there is a need for more visible AE&T, the usability can become challenging as the disability is now visible to others:

It’s almost like being diagnosed again. They often need a bit of support to work out how they fit it into their lives. (Yumi)

The emotional aspect related to AE&T has multiple layers in the participants’ responses. Sunita and Yumi’s discourse suggests a potential conflict between AE&T (meaning the disability associated with MS) and the person’s identity. In this sense, accepting change by introducing an AE&T represents their disability and further confirms their condition:

There’s a drive to not let the MS win. (Sunita)

When you start to mention wheelchairs or stairlifts to people, what they’re hearing is now the MS has beaten you, I have become disabled. (Yumi)

Some will perceive it more openly thinking about majority people, though it’s never easy to accept new disability. So, there is a certain perception of accepting equipment as accepting disability. Because it’s another indicator of kind of progression of the condition. (Sam)

Beyond technology are the characteristics of the MS condition and how individuals perceive the impact on their occupations. Sunita described how MS might influence what individuals can do by themselves. At the same time, Tarik pointed out that not being able to participate in meaningful occupations is an aspect that can lead to AE&T usability:

I think it depends on the stage of the condition that someone is at. (Sunita)

I think that’s just something you’re just not going to be able to do any more, there’s not an option really. (Tarik)

Therefore, technology also has an additional significant meaning related to turning points in life when individuals cannot participate in occupations associated with MS stages. Tarik shared a situation involving a mother who rejected AE&T until she became frustrated and could no longer walk their child to school or play with them in the park:

She said to me, right, buy one. I said what, and she said, I get it, I get what this gives me, I can get to the park with my son now, okay, I can’t play football with him when we’re there, but I can watch him on the swings, and I can see how this makes me more able now.

Theme 2 stresses how in addition to cognition, meaning and emotional value show the complexity of involving AE&T acceptance and usability. Furthermore, it is related to how individuals possibly see technology as a signal of declining or appearing to lose their identity. In this direction, Themes 1 and 2 present technology’s cognitive challenges and meaning, respectively. Theme 3 will describe how occupational therapists considered the person before technology and some of their strategies to engage individuals in technology use.

### Theme 3: ‘They have to experience it by doing’

Across Theme 3, participants described their practices using different approaches to support people with MS to become more receptive to AE&T. These practices were expressed by prioritising the person’s history and needs. Yumi suggested motivational interviewing as an approach to encourage people to establish and meet their goals, where AE&T can perhaps help them achieve these goals.

So, you might not get them to the point where they’re saying, ‘yeah, I’m definitely going to do this’, but you get them to a point where they’re further down the line of the readiness to change and that can be really helpful. (Yumi)

Sam communicated how the doing underlies the assessment process. Leili described using a graded approach that involves gradually introducing simple to more complex AE&T, adapting goals and the occupational experience with realistic and achievable use of technology. Both discourses exemplify the uniqueness of occupational therapy reasoning focused on occupational participation:

Again, doing those kind of comprehensive assessments, looking at other factors skin integrity, incontinence, ability to move, position, method of transfer that you kind of take into account and what that seating is going to be used for, what’s the function of that seating? What is the individual going to be doing from that? (Sam)

You start with something that’s graded down. So, they’re successful with that effort and then you add more complexity, or different goals. So, you’re adding more goals as you go along. If we can introduce something, it has to work from the off. So, it has to be simple enough that they could operate without effort in a way [. . .]. So, in a way, they have to experience it by doing it and then they can see that it works. (Leili)

This quotation suggests that facilitating individuals with MS to physical trial and practice using AE&T can be an opportunity for them to experience the benefits and better understand its use. Yumi also suggested another perspective regarding the power of peer support groups in facilitating the acceptance and usability of AE&T. This description shows that people with MS might often connect with others who share similar experiences, which can encourage the usability of technology.

She’d never taken it up and then somebody else with MS recommends it and she’s doing it. (Yumi)

Sam and Sunita’s discourse illustrates the power-balanced relationship between the occupational therapist and the person. The first one is not seen as the expert who will prescribe technology and make the decisions; instead, they consider individuals with MS the experts of their own lives.

I always say to them ‘you’re the expert in MS’, you know, I know a bit about it, but I look to them to say, right, what do you want? (Sam)

Those that are further down the line are the expert patients, they know their condition. They might not be willing to accept it’s changing but you can still take on board exactly what their wishes are and try and understand how it will affect their family life, their home life. . /. (Sunita)

Sunita’s lived experience working with individuals with MS demonstrates that AE&T cannot be the main focus of therapy. Once they do not accept changes, it is still a place to explore priorities, and what matters to them so that occupational therapists can understand their needs and meaningful relationships.

The participants placed the person before technology so that AE&T acceptance and usability are related to the person’s occupational lives and relationships. Yumi suggested that AE&T acceptance and usability require therapists to employ an array of skills such as building rapport, active listening to the person, family and carers, using effective communication strategies, having an open conversation about risks, providing reassurance and considering time and choice. All these approaches consist of actively listening to individuals’ occupational needs and the involved relationships:

We have to use this equipment to compensate for that loss. So again, it’s choosing the right time. Choosing the right place and choosing the right people to be there are really important. It’s not something you can literally just walk up in a very quick session and say ‘oh by the way, you can’t now use that stand aid, you need to move to a hoist’. (Sam)

The most important thing you can do is to listen to people and to work out what it is they want to do, need to do and have to do. (Yumi)

So, it’s listening to them involving their families as well and carers. (Sunita)

The three themes are intertwined to explore the complexity of AE&T in the lives of individuals with MS. The participants expressed aspects of cognition and emotional value associated with technology. Conversely, as Leili, Yumi, and Sunita stated in Theme 3, occupational therapists’ role when working with individuals with MS and AE&T is to prioritise client-centred occupation.

## Discussion

Our study investigated occupational therapists’ perceptions regarding the acceptability and usability of AE&T by individuals with MS. Our findings revealed that cognitive changes in individuals with MS, the meaning of technology and person-centred care are elements related to acceptability and usability, informing the holistic approach of the occupational therapists interviewed.

In Theme 1, participants described that adults with MS can have difficulty adapting to use new pieces of AE&T because it requires them to learn strategies, which can be challenging with a cognitive impairment; this could be one of the possible reasons why individuals with MS might not accept and use AE&T. Hedman et al. (2017) and Cruz et al. (2016) shared a similar finding that people with cognitive impairments struggle to learn how to use AE&T resulting in technology abandonment. Yet, our research findings suggest that although participants emphasised the cognitive aspect, they did not reduce this component to the cause-effect of acceptance and usability. Rather, it appears that the complexity of MS is related to the progressive characteristic of the disease, which demands individuals’ adaptive skills to deal with the challenges of their daily lives, as described by Yumi and Sunita. It is also known that individuals with MS who lack problem-focused coping and/or fail to seek social support have less psychological adjustment regarding the MS condition (McCabe et al., 2004).

Our findings also show that some individuals with MS do not accept technology until they lose their ability to sustain their occupational participation without a device. Interestingly, current concepts of occupational participation do not restrict the term to merely the act of doing, but to access, initiate and sustain occupations that are valued in meaningful contexts and relationships (Egan and Restall, 2022). The concept echoes that occupational therapists have a role in exploring the initiation, access and sustainability of occupational participation with individuals with MS, for example, testing approaches such as the Cognitive Orientation to Daily Occupational Performance – CO-OP Approach^TM^, which has demonstrated evidence in improving occupational performance in individuals with MS (Saeidi Borujeni et al., 2023).

Theme 2 explored the meaning behind technology. Participants shared their lived experiences working with individuals with MS who report different meanings. First, technology seems to represent losing skills, a process of degeneration, having a disability and associated with stigma, as pointed out by Yumi and Sunita. In this sense, AE&T offers possibly visual representations of disability that force individuals to adapt and adopt a new way of living that they did not plan for themselves, for example, accepting their future as a disabled person when they are still trying to reflect and adapt psychologically to the changes that MS has caused. Moreover, evidence from studies by Souza et al. (2010) and Squires et al. (2019) identified that even with the benefits, technology could be seen as a symbol of disability by users with MS. In particular, a study conducted in the UK reported that individuals expressed different experiences and feelings when they used technology, such as receiving ‘funny looks’ and ‘feeling invisible’ (Squires et al., 2019: 484).

Likewise, it can be argued that the nature of MS presents turning points marked by progressive transitions that bring uncertainty about the future. According to McCabe et al. (2004), the unpredictable development of MS elicits a great deal of uncertainty regarding the future health and well-being of individuals. A synthesis of qualitative evidence of individuals with MS lived experience also identified that uncertainty disrupted their routine, values roles and activities (Desborough et al., 2020).

On the other hand, technology also means opportunities for occupational engagement. As described by Tarik, the story of a mother who could not play football with her son in the park, even with technology, was still unable to play physically, but being in that environment connected to her son through the occupation, gave her the feeling of being able to engage. In this case, occupational engagement is illustrated by essential or influential elements: a sense of readiness, meaning, motivation, interest and a supportive environment (Kennedy and Davis, 2017). In addition, instead of the ‘doing’ dimension of occupation, technology can also contribute to sustaining the ‘being’ aspects of individuals (Wilcock, 2006).

In Theme 3, occupational therapists shared their expertise working with individuals with MS to facilitate acceptance and usability of AE&T. It was clear that their approach did not restrict the role of occupational therapists to ‘technology prescribers’. Congruent with occupational therapy values, the participants’ approaches aimed, first, to understand the individuals’ occupational needs, in line with the definition of the World Federation of Occupational Therapists (2012) that states the outcomes of health and well-being can be achieved through occupation to facilitate occupational engagement. In our research, occupational therapists described challenges beyond cognitive impairment also related to the occupational identity of clients with MS. They expressed how acceptance and usability of AE&T need consideration of who individuals were in their past, their current *status* of occupational participation and what they could do to enable participation by technology introduction. Participants also referred to turning points in the people with MS’s lives alongside the need to engage with others (husband, children), leading to acceptance of the technology, particularly as they could identify the meaning and rationale.

A person-centred approach was remarkably evident in occupational therapists’ discourse by referring to eliciting individuals’ active participation in the therapeutic process; for example, through motivational interviews, goal formulation and grading goals, occupational therapists revealed their strategies to engage individuals with MS regarding AE&T, considering them as experts of their own lives. These strategies locate the person’s needs as central to occupational therapy practice to define the plan together, maintaining emphasis on everyday life (Kos et al., 2023).

Interestingly, Leili described specific strategies. Since individuals with MS can present with symptoms of fatigue, depression, anxiety and different cognitive difficulties (Ponzio et al., 2023), a graded approach using technologies with varied levels of complexity could work as an element of engagement with technology. A graded approach is well evidenced in occupational therapy practice, and it is a result of an occupational analysis, which can also equip occupational therapists to support individuals with cognitive impairments to achieve sequential tasks of an occupation (Giles et al., 2019). Moreover, Squires et al. (2019) identified that individuals with MS can benefit from this grading approach when the condition progresses; thus, technology can be associated with a hierarchy of basic to more complex equipment.

Yumi described peer support as an important approach because individuals with MS could share their experiences with people living with the same condition. This approach underlines the social environment’s role in mediating the acceptance and use of technology. Furthermore, Squires et al. (2019) also corroborate that supportive social networks from families and carers can promote acceptance and the use of AE&T. The strategy of Yumi is also evidenced in a meta-synthesis study focused on the lived experience of individuals with MS. Adapting life was seen as beneficial from the engagement with others living with the same condition (Desborough et al., 2020).

Sunita shared the importance of facilitating opportunities for individuals with MS to experience the equipment. We can speculate that trying the technology and having experience using it could help individuals with MS perceive its benefits in their daily lives and offer them increased control and choices. These examples resonate with the holistic approach of occupational therapists rooted in the relationship between the person, occupation and environment and how technology can facilitate participation in life roles (Steel et al., 2017).

The matching person and technology (MPT) (Scherer, 2017) and the human, activity and assistive technology (HAAT; Cook et al., 2019) are the most cited person-centred conceptual models of assistive technology in the literature (Alves and Matsukura, 2016). The HAAT model equips therapists to describe the person who engages in occupations (activities) and their need to use technology within a context, therefore, not focused on the assistive technology device in itself, guiding service delivery, evaluation and research (Cook et al., 2019). In the MPT model, attention should be focused on the environment in which the technology will be utilised, aligned with the person’s preferences and needs, and the characteristics of technology (Scherer, 2017).

Both models consider the importance of the person’s experience with the equipment within a holistic context. As occupational therapists use occupations to support occupational engagement, an occupation-centred practice is expected to facilitate the individual experience of occupational participation with an AE&T. However, in our research, participants interviewed did not refer to any conceptual models or standardised assessments of assistive technology to facilitate acceptance and usability of AE&T. Perhaps, because the focus of the interviews was not on the assessment or the service delivery objectively. Moreover, in the UK, it is unknown whether these models are utilised by practitioners in the National Health Service – NHS (Tedesco Triccas et al., 2019). Since specific models of assistive technology can contribute to evidence-based practice, future studies could address whether occupational therapists use these models in their practices.

## Limitations

As limitations, our study considered the subjectivity of the participants’ position as it relied upon their perspectives and recollections. Consequently, these could have been assumptions rather than specific conversations they had with people with MS and their AE&T. Despite that, our research provides a true reflection of five experienced skilled occupational therapists’ views, similar to a previous study with four occupational therapists with extensive experience with MS and AE&T in the UK (Squires et al., 2019). In further research, it would be beneficial to conduct a similar study comprising individuals with MS, their families and carers to illuminate their lived experiences. The transferability of our research is difficult to determine. However, the findings presented are reflected in much of the existing literature.

## Conclusion

Our study has utilised a qualitative descriptive approach to understand better the acceptance and usability of AE&T by individuals with MS from occupational therapists’ perspectives. Transitions related to the nature of MS, such as changes in cognition, and adaptive skills, were seen as factors related to the acceptance and usability of AE&T. The meaning of technology was discussed as another influential element with multiple layers representing disability, losing abilities and deterioration; however, turning points in life and the need for occupational participation also appeared to influence technology acceptance and usability. According to participants’ responses, occupational therapy approaches were both person- and occupation-centred. They included approaches such as grading technology and goals, experiencing the doing with the technology, peer support and acknowledging the individuals with MS as experts in their own lives. Therefore, our findings highlight the importance of using core occupational therapy principles and approaches to best support people with MS in using AE&T effectively.

**Key findings**

- Cognitive challenges are perceived by occupational therapists as a critical element in adaptation, acceptability and usability of AE&T by individuals with MS.
- The meaning of technology can influence the acceptability and usability of AE&T by individuals with MS.

**What the study has added**

Occupational therapists’ perception of AE&T shows that a complex holistic approach is essential to enable acceptance and usability.
